# General and family medicine physicians’ perception of the concept of good death: a contribution to the validation of the scale

**DOI:** 10.1186/s12875-025-03113-4

**Published:** 2025-12-01

**Authors:** Sofia Carvalheiras, Andreia Teixeira, Ivone Duarte

**Affiliations:** 1 Faculty of Medicine, University of Porto, Porto, 4200-450 Portugal; 2 Santa Luzia Family Health Unit, Tâmega e Sousa Local Health Unit, Porto, Portugal; 3 Department of Community Medicine, Health Information and Decision-Making, Faculty of Medicine, RISE-Health, University of Porto, Alameda Prof. Hernâni Monteiro, Porto, 4200-319 Portugal; 4 Center of Bioethics of the Faculty of Medicine, University of Porto, Porto, 4200-450 Portugal

**Keywords:** Cross-Sectional studies, Family practice, General practitioners, Terminal care, And validation study

## Abstract

**Background:**

General and Family Medicine (GFM) physicians play a key role in end-of-life care, yet there is limited knowledge about how they understand and value the components of a good death in their clinical practice. The Concept of a Good Death scale, originally developed by Schwartz et al., offers a validated instrument to explore its meaning. The present study aims to conduct a psychometric validation of the Portuguese version of the Concept of a Good Death instrument among GFM physicians in Portugal.

**Methods:**

A cross-sectional methodological validation study was conducted between December 2024 and February 2025 using an online questionnaire. The study sample consists of 200 GFM physicians. The questionnaire included sociodemographic questions, previous experience with illness or death, and the 17-item Concept of a Good Death scale. Principal component analysis was performed to explore the factor structure. Internal consistency was assessed using Cronbach’s alpha.

**Results:**

Among the 200 participants initially recruited, one participant refused to provide informed consent after reading the study presentation, and another submitted an incomplete response, omitting most of the instrument’s items. These two cases were excluded from the analyses, resulting in a final sample of 198 physicians, corresponding to a response rate of 99%. Principal component analysis supported a three-factor model (Closure, Personal Control, and Clinical domains) accounting for 53% of the total variance. The overall internal consistency of the scale was good (α = 0.839). The factor structure was generally consistent with the original version, except for one item, potentially reflecting the clinical perspective of the sample.

**Conclusions:**

The Portuguese version of the Concept of a Good Death scale demonstrates good psychometric properties among GFM physicians. Its application may support better understanding of healthcare professionals’ perceptions of a good death, promote more person-centered end-of-life care and contribute to palliative care education. Further studies are recommended to validate the scale across other professional and cultural groups and to strengthen its validation through approaches such as confirmatory factor analysis.

**Supplementary Information:**

The online version contains supplementary material available at 10.1186/s12875-025-03113-4.

## Background

If there is a non-negotiable right to life, there is also a right to a good death. The concept of a good death is individual, complex and multifaceted, changing over time, and influenced by cultural, religious, social, and clinical factors [1–4].

In the pre-modern era, a good death was primarily understood in spiritual terms, emphasizing reconciliation with God and the presence of family at home. With modernity, death came to be seen as a failure of medicine, often hidden or postponed, with the hospital becoming the predominant place of death. In contemporary times, influenced by palliative care and the debate on euthanasia, a good death is recognized as an integral part of life, with particular emphasis on control over the dying process and symptom management [5].

Although there is some consensus on the characteristics of a good death, the importance attributed to each one varies among patients, families, and healthcare professionals, reflecting its complexity and subjectivity [1]. Death is still frequently perceived in healthcare services as something uncomfortable, and the lack of reflection on finitude can lead to a technical and impersonal approach, prolongation of life without real clinical benefit, and additional suffering for patients, families, and professionals [6–8]. In this context, reflecting on death is fundamental for ethical and humanistic medical practice.

This reflection is especially relevant in General and Family Medicine (GFM), as proximity to the community, continuity of care, and trust-based relationships with patients place family physicians at the forefront of end-of-life care, making them key interlocutors in ensuring dignified deaths aligned with patients’ values [9–11]. However, the conceptions of these professionals regarding the dying process remain underexplored, shaped largely by clinical practice and institutional context.

Schwartz et al. [12] developed a study with the objective of measuring the importance of several components considered critical to the concept of a good death, according to medical students, nursing students, students of disciplines outside the health area and nurses working in palliative care. In other words, it aims to assess the importance attributed to different components from the individual’s own point of view [12]. The work was developed based on Walden-Galuszko’s [13] concepts, such as total suffering (physical, spiritual, social and psychological) and spirituality. The Concept of a Good Death instrument presents three domains. The Closure domain reflects psychosocial and spiritual aspects of a good death, being associated with beliefs about life after death, with social support, as well as with spiritual practices and convictions. The Personal Control domain focuses on the physical aspects of the death experience, mental alertness, communication skills, and control of bodily functions. The Clinical domain refers to the clinical and biomedical factors of death and is generally associated with the perception of death as a relief. The measure appears to be reliable and valid in the study carried out by the authors, having the advantage of being brief, self-completion and being able to be used in a wide variety of populations. According to the authors, it can be used by healthcare providers and lay people in the context of research, with the aim of improving the quality of care provided at the end of life and evaluating the impact of the medical curriculum on teaching palliative care.

Given the importance of understanding the perception of a good death in clinical practice and ensuring end-of-life care aligned with patients’ values, it is essential to have psychometrically valid and culturally adapted instruments. In this context, a study was carried out to validate the Concept of a Good Death scale for the Portuguese population [14]. This study [14] involved a sample of 100 participants and followed the methodological procedures of translation, back-translation and cultural adaptation, with authorization from the original author. This validation [14] was conducted on a sample composed of students and health professionals, without focusing on different clinical groups.

Considering the central role of these professionals in end-of-life care and the growing need for humanized care, the present study aimed to conduct a psychometric validation of the Portuguese version of the Concept of a Good Death instrument among GFM physicians in Portugal.

## Methods

### Study design

We conducted a cross-sectional methodological validation study using an online questionnaire via Google Forms^®^ among GFM physicians in Portugal. This design was chosen because cross-sectional studies are appropriate for psychometric validation, allowing assessment of construct validity, internal consistency, and factor structure at a single point in time without intervention. Online surveys facilitate efficient data collection across a geographically dispersed population while ensuring anonymity and voluntary participation. This approach follows recommended practices for instrument and scale development, as outlined by Streiner and Kottner [15], whose reporting guideline is endorsed by the EQUATOR Network, and is consistent with methodologies commonly employed in similar validation studies.

### Questionnaire

The questionnaire consisted of three sections: (a) sociodemographic information, (b) previous experience with illness or death, and (c) the Concept of a Good Death measure [see Additional file 1].

The instrument used was the Portuguese version of the Concept of Good Death scale, originally developed by Schwartz et al. [12] and subsequently translated and culturally adapted by Jordão and Leal [14]. Use of this version was based on prior authorization obtained from the original authors, which already covered application of the adapted version, and therefore no additional permission was requested from either the original authors or the adaptors.

### The concept of a good death measure

The Concept of a Good Death measure consists of 17 items, assessed on a 4-point Likert scale, where 1 corresponds to “Not necessary,” 2 to “Desirable,” 3 to “Important,” and 4 to “Essential.” The instructions for completing the scale were: “Please indicate how important each of the following items is to your conception of a “good death”. The scale items are organized into three distinct factors: Closure (items 4, 6 to 13), Control (items 15 to 17), and Clinical (items 1 to 3, 5, and 14). The score for each factor was calculated by summing the scores of the corresponding items, as defined by the authors of the original scale. This approach allows for a total score per factor, reflecting the degree of importance attributed to each domain of the good death concept. In the original version of the scale, the authors reported internal consistency values ​​(Cronbach’s alpha) of 0.75 for the Closure factor, 0.83 for the Control factor and 0.62 for the Clinical factor [12].

### The participants

The study population consisted of GFM physicians practicing in Portugal. Eligible participants were either specialists or residents in GFM. No exclusion criteria were defined, so all physicians meeting the inclusion criteria and completing the online questionnaire were included in the study. Recruitment followed a non-probabilistic convenience sampling strategy.

Eligible physicians were invited to participate by completing the online questionnaire. The survey was distributed through institutional email lists of Family Health Units (USFs) and Personalized Healthcare Units (UCSPs) in Portugal, in collaboration with the College of GFM of the Portuguese Medical Association, as well as via other digital platforms, such as professional WhatsApp^®^ groups. Prior to accessing the questionnaire, participants were asked to provide informed consent. This was obtained electronically through a mandatory multiple-choice item (“yes” or “no”), and only those who selected “yes” were allowed to proceed.

Data collection was carried out between December 2024 and February 2025. Responses were automatically stored in a secure Google^®^ Spreadsheet database accessible only to the authors, with no identifying information collected. All data were anonymized prior to analysis and were used exclusively for research purposes. The confidentiality and anonymity of participants were strictly maintained in accordance with ethical standards for research involving human participants.

### Sample size

Principal component analysis is sensitive to sample size, as small samples can compromise the stability of the extracted factors and the reliability of factor saturations. Even so, there is no absolute consensus in the literature regarding the ideal number of participants. We chose to follow the guidelines of Comrey [16] and Kline [17], leading authors in the field of psychometrics, who indicate that a sample of 200 individuals is considered “good” for exploratory factor analysis (EFA). A total of 200 GFM physicians, recruited through convenience sampling, participated in the scale validation study; therefore, our sample size satisfies these recommendations.

### Statistical methods

Data analysis used statistical software SPSS Statistics V.28. Descriptive statistics were used to characterize the sample, using the absolute and relative frequencies, n (%). To compare proportions in distributions, the binomial test was applied. To characterize the distribution of responses to Likert-type scale items, measures of central tendency and dispersion were calculated, namely, mean, standard deviation and median. For the validation process, principal component analysis was applied to examine the internal structure of the scale. Varimax rotation was used, and a minimum factor loading threshold of 0.4 was applied to retain items. The Kaiser-Meyer-Olkin (KMO) measure and Bartlett’s test of sphericity were used to assess sample adequacy. The recommendations indicate that KMO should have values higher than 0.6 to apply the principal component analysis [17]. The internal consistency of the scale was evaluated using three techniques: interitem correlation (mean of the interitem correlations), corrected item–total correlation, and Cronbach’s alpha. Following Kline [17], a mean interitem correlation above 0.4 was considered indicative of adequate internal consistency. Corrected item–total correlations were interpreted according to Kline’s recommendations [17] as follows: 0.4–1.0 = very good, 0.3–0.39 = good, 0.2–0.29 = sufficient but needs improvement, and < 0.2 = weak, reject or revise. Cronbach’s alpha was interpreted according to commonly accepted thresholds, as described by Hill and Hill [18]: >0.90 = excellent, 0.80–0.90 = good, 0.70–0.79 = acceptable, 0.60–0.69 = questionable, and < 0.60 = unacceptable.”

### Pre-test

Before the final application of the questionnaire, a pre-test was conducted with a group of approximately 15 GFM physicians, selected by convenience by the study authors. The main objective of this stage was to assess the clarity and comprehension of the items, as well as to estimate the average time required to complete the instrument. This phase also allowed the identification of potential difficulties in interpretation, redundancies, or ambiguities in the questions, enabling adjustments and ensuring that the final questionnaire was suitable and functional for the target population. The average response time was approximately 3 minutes, and no modifications were required.

### Ethics

The study protocol was approved by the Ethics Committee of the Faculty of Medicine of the University of Porto, Reference No. 274/CEFMUP/2024, on September 27, 2024, and followed the ethical principles outlined in the Declaration of Helsinki [19]. In accordance with these principles, all participants provided their informed and voluntary consent electronically.

## Results

### Sample

A total of 200 physicians were initially recruited. One participant declined to provide informed consent after reading the study presentation, and another submitted an incomplete response, omitting a large portion of the instrument’s items. These two cases were excluded from the analyses, resulting in a final sample of 198 physicians, corresponding to a response rate of 99% (Table 1). Missing data for specific variables were duly recorded and marked. The sociodemographic characteristics of the sample, along with information on previous experiences with illness or death, are presented in Table 1.

**Table 1:** Participants’ characteristics

Variables	n (%)
Sex	
Female	157 (79.3)
Male	41 (20.7)
Nationality	
Portuguese	193 (97.5)
Other	5 (2.5)
Ethnicity (*n* = 196)	
Caucasian	192 (98.0)
Black	3 (1.5)
Asian	0 (0)
Other	1 (0.5)
Age range	
Less than 30 years	28 (14.1)
Between 30 and 39 years	94 (47.5)
Between 40 and 49 years	48 (24.2)
Between 50 and 59 years	13 (6.6)
Between 60 and 69 years	13 (6.6)
At least 70 years	2 (1.0)
Workplace region	
North	97 (49.0)
Center	30 (15.2)
West and Tagus Valley	18 (9.1)
Lisbon Metropolitan Area	34 (17.2)
Alentejo	3 (1.5)
Algarve	5 (2.5)
Autonomous Region of the Azores	7 (3.5)
Autonomous Region of Madeira	4 (2.0)
Professional category (*n* = 196)	
Specialist	146 (74.5)
Resident	50 (25.5)
Number of years of professional experience	
Between 1 and 10 years	102 (51.5)
Between 11 and 20 years	58 (29.3)
Between 21 and 30 years	25 (12.6)
Between 31 and 40 years	6 (3.0)
More than 40 years	7 (3.5)
I have a chronic illness	
Yes	52 (26.3)
No	146 (73.7)
I have been seriously ill	
Yes	31 (15.7)
No	166 (84.3)
I dealt with the death of a patient	
Yes	183 (92.4)
No	15 (7.6)
I dealt with the death of someone close to me	
Yes	181 (91.4)
No	17 (8.6)

Most participants are female (79.3%), of Portuguese nationality (97.5%) and of Caucasian ethnicity (98.0%), with the most frequent age group being between 30 and 39 years old (47,5%). The majority of participants are specialist doctors (74.5%) and have between one and ten years of professional experience (51.5%), working in the North of Portugal (49.0%). Most participants do not have a chronic disease (73.7%) and have never been seriously ill (84.3%). Regarding previous experience with death, a large part of the sample had already dealt with the death of a patient (92.4%), as well as the death of someone close (91.4%).

When compared with the population of GFM physicians registered in the Portuguese Medical Association up to December 2024, our sample is not representative in terms of sex (*p*-value < 0.001). Regarding age, a direct comparison was not possible due to differences in the grouping of age categories. However, a comparative overview of the sample and population distributions for both sex and age is provided in an additional figure [see Additional file 2].

### Descriptive analysis of the scale

Item means ranged from 1.47 (SD = 0.85) to 3.73 (SD = 0.59), indicating a diversity of perceptions regarding the content of the items. Medians ranged from 1 to 4, with interquartile values ​​revealing different patterns of response concentration. Items such as “That it be painless or largely pain-free” (M = 3.73; SD = 0.59) and “That it be peaceful” (M = 3.67; SD = 0.62) obtained higher means, suggesting greater agreement on the part of the participants. In contrast, items such as “That death occurs during sleep” (M = 1.88; SD = 0.91) and “That there be mental alertness until the end” (M = 1.98; SD = 0.92)) presented lower means, indicating less consensus or importance attributed. The dispersion of responses, analysed through standard deviation, varied between 0.59 and 0.95, revealing moderate variability in responses. Items with greater variability included “That the dying period be short” (SD = 0.95) and “That the person was able to remain at home” (SD = 0.93) [see Additional file 3].

### Factor analysis

The scarp plot corroborates the suggestion of extraction into 3 factors, which corresponds to the structure of the original scale, so a 3-factor analysis was performed using the principal components method (Table 2). The KMO measure revealed a medium sampling adequacy (KMO = 0.795) and Bartlett’s sphericity test rejected the null hypothesis of no correlation between the variables (*p* < 0.001). In addition, the diagonal values ​​of the anti-image matrix are all greater than 0.5 and the values ​​below the diagonal are mostly low. The 3 factors explain 53% of the total variance.

After applying a rotation using the varimax method, item saturation was obtained. The first factor explains 29.3% of the total variance and consists of 10 items (1, 4, 6–13). The second factor explains 13.5% of the total variance and consists of 3 items (15, 16, 17). The third factor consists of 4 items (2, 3, 5 and 14) and explains 10.4% of the total variance.

**Table 2:** 3-factor factor analysis (values not in bold represent the loadings before rotation; values in bold represent the loadings after rotation)

	Factor 1	Factor 2	Factor 3
That it be painless or largely pain-free.	**0.548**		0.485
That the dying period be short.			**0.799**/0.669
That it be sudden and unexpected.			**0.763**/0.525
That family and doctors follow the person’s wishes.	**0.645**/0.525		
That it occur naturally, without technical equipment.		0.395	**0.420**
That it be peaceful.	**0.649**/0.510		
That loved ones be present.	**0.481**/0.540		
That the person’s spiritual needs be met.	**0.756**/0.555		
That the person is able to accept death.	**0.533**/0.635		
That the person had a chance to complete important tasks. (*n* = 197)	**0.691**/0.634		
That the person had an opportunity to say “good-bye”. (*n* = 197)	**0.765**/0.700		
That the person was able to remain at home.	**0.409**/0.534		
That the person lived until a key event.	**0.581**/0.663		
That death occurs during sleep.	0.507		**0.703**
That there be mental alertness until the end.	0.595	**0.829**	
That there be control of bodily functions until death.		**0.792**/0.568	
That the ability to communicate be present until death.	0.635	**0.806**	

### Internal consistency analysis

Cronbach’s alpha applied to the 17 items is 0.839, which indicates good reliability.

Table 3 shows the reliability by factor. In factor 1, Cronbach's alpha is good (α = 0,826), and removing no items improves its value. All items, except for item 1, have very good item-total correlation. Item 1 has good item-total correlation. The average inter-item correlation is moderate. In factor 2, Cronbach's alpha is good (α = 0,867), and removing no items improves its value. All items show very good item-total correlation. The average inter-item correlation is good. In factor 3, Cronbach's alpha is reasonable (α = 0,700), and removing item 5 would improve its value. All items, apart from item 5, have very good item-total correlation. Item 5 has good item-total correlation. The average inter-item correlation is moderate.

**Table 3:** Internal consistency analysis

	Cronbach’s Alpha if item is deleted	Corrected item-total correlation	Average inter-item correlation
Factor 1 (α = 0.826)			0.331
1. That it be painless or largely pain-free.	0.823	0.374	
4. That family and doctors follow the person’s wishes.	0.813	0.495	
6. That it be peaceful.	0.814	0.490	
7. That loved ones be present.	0.821	0.441	
8. That the person’s spiritual needs be met.	0.803	0.601	
9. That the person is able to accept death.	0.812	0.505	
10. That the person had a chance to complete important tasks.	0.801	0.609	
11. That the person had an opportunity to say “good-bye”.	0.794	0.683	
12. That the person was able to remain at home.	0.821	0.425	
13. That the person lived until a key event.	0.805	0.567	
Factor 2 (α = 0.867)			0.686
15. That there be mental alertness until the end.	0.808	0.752	
16. That there be control of bodily functions until death.	0.819	0.741	
17. That the ability to communicate be present until death.	0.811	0.748	
Factor 3 (α = 0.700)			0.370
2. That the dying period be short.	0.643	0.474	
3. That it be sudden and unexpected.	0.583	0.573	
5. That it occur naturally, without technical equipment.	0.720	0.342	
14. That death occurs during sleep.	0.584	0.564	

## Discussion

The validation results of the instrument show that the Concept of a Good Death scale consists of 17 items. EFA revealed a three-factor structure, similar to that proposed in the original study by Schwartz et al. [12], corresponding to three distinct dimensions of the concept of a good death. The original factor labels were retained given the similarity of the data obtained.

The first factor, consisting of items 1, 4, and 6 to 13, was named Closure, reflecting psychosocial and spiritual aspects of a good death. The second factor, named Control, included items 15 to 17. This designation reflects the focus of this dimension on the physical aspects of the dying experience, such as control of bodily functions, communication ability, and mental alertness. The third factor, consisting of items 2, 3, 5, and 14, was designated Clinical and refers to the clinical and biomedical factors of death. Altogether, the three factors explained 53% of the total variance.

Regarding the distribution of items across factors, compared with the original scale, the difference lies in the allocation of item 1, “That it be painless or largely pain-free”. In the present study, this item loaded on the Closure factor, while in the original study it loaded on the Clinical factor. A possible explanation is the frequent conceptual confusion between pain and suffering. Although they are distinct, insufficient differentiation may lead to confusion in both clinical practice and theoretical reflection [20]. Pain is a physical sensation, measurable and treatable, whereas suffering is a subjective, unique, and individual experience related to a threat to a person’s integrity—not only of the body, but also of identity, social roles, hope, and sense of meaning in life [21, 22]. According to Eric Cassell, suffering is not limited to physical pain but also encompasses social, spiritual, and psychological aspects, and may exist even in the absence of pain, such as in the loss of a loved one [21, 23]. Moreover, the original study sample consisted mainly of students, whereas the present study was conducted among physicians. Recognizing suffering requires an empathic, person-centered attitude, active listening, and an understanding of the patient’s emotional and existential context [22]. These skills can be developed through daily and frequent contact with patients, enabling physicians to become more aware of the multidimensional nature of suffering and to recognize aspects that go beyond physical pain. Since item 1 does not explicitly mention “physical pain,” confusion between the two concepts may have occurred, influencing the interpretation of the scale by the physician sample, who are more aware of all dimensions of suffering.

The descriptive analysis showed that the scale is sensitive in capturing different perspectives on the concept of a good death, with mean scores ranging from low values (M = 1.47; SD = 0.85) to high values (M = 3.73; SD = 0.59) on a 1-to-4 scale. These results indicate that some items were considered less relevant while others were regarded as highly important by participants. Items such as “That it be painless or largely pain-free”, “That it be peaceful,” and “That family and doctors follow the person’s wishes.” obtained high mean values. By contrast, items such as “That death occurs during sleep” and “That there be lucidity until the end” presented lower mean values.

The validation study revealed satisfactory Cronbach’s alpha values for the three factors. Closure (α = 0.826) and Control (α = 0.867) demonstrated good internal consistency, while Clinical (α = 0.700) showed acceptable consistency. In the original version of the scale [12], the authors reported α = 0.75 for Closure, α = 0.83 for Control, and α = 0.62 for Clinical. The comparison highlights slightly higher internal consistency in all factors in the present study, particularly for Clinical, which improved from weak to acceptable.

In the case of the third factor, removal of item 5, “That it occurs naturally, without medical equipment,” would improve the Cronbach’s alpha value. It is important to note that this item, although included in the Clinical dimension of the concept of a good death, presents characteristics that may explain its weaker correlation with the other items in the factor and, consequently, the fact that its removal increases Cronbach’s alpha. While items 2, 3, and 14 emphasize aspects related to speed, unpredictability, and the possibility of a peaceful death without awareness of the moment, features centered on the immediate experience of dying, item 5 refers to the presence or absence of medical intervention and how the dying process is technically managed. This focus on the “natural” character of death, without technological support, may evoke ethical or philosophical considerations different from those captured by the other items, thus contributing to weaker statistical coherence, although it remains conceptually relevant. Furthermore, this is a potentially ambiguous item, open to multiple interpretations by participants. The expression “naturally, without medical equipment” may be understood in various ways: as a refusal of palliative care, as an ideal of death occurring at home, or even as a critique of the medicalization of dying. These different interpretations may have led participants to respond less consistently to item 5 compared with other items in the same factor, which may explain its weaker correlation with the factor and consequent impact on Cronbach’s alpha. Nevertheless, despite these limitations, we opted to retain the item in the scale, ensuring that it captures not only the more consensual dimensions but also the more controversial and context-dependent aspects of the concept of a good death.

Although the present study provides preliminary evidence of the validity and reliability of the Portuguese adaptation of the *Concept of a Good Death* scale, some methodological limitations should be noted. Only EFA was conducted, given that this was the first application of the instrument in general and family medicine physicians in Portugal and no prior validation data were available. Confirmatory factor analysis (CFA) would require an independent sample, which was not available; therefore, it is recommended for future studies to replicate and confirm the factorial structure [24, 25]. Other forms of validity testing, such as predictive, concurrent, convergent, and discriminant validity, were not performed in this initial study and should be explored in future research to further strengthen the psychometric evidence for the scale. The exclusive focus on general and family medicine physicians limits the generalizability of the findings to other specialties and cultural contexts. Furthermore, the sample is not fully representative of the population of GFM physicians in Portugal, particularly regarding sex distribution, which may limit the external validity of the results. Moreover, the cross-sectional design did not allow for the assessment of temporal stability, and test–retest reliability could not be performed since participants were only invited to complete the questionnaire once. Additional limitations include the use of digital data collection, which may have introduced selection bias, and the presence of some missing responses.

Future research is suggested to explore the application of this scale in other professional and cultural groups, thereby contributing to a broader understanding of the concept of a Good Death. Further studies should also include CFA to replicate and strengthen the factorial structure identified in this study, as well as test–retest reliability to assess the temporal stability of the instrument. Additionally, future work should examine predictive, concurrent, convergent, and discriminant validity to provide a more comprehensive validation of the instrument. Finally, it is recommended that item 1 be reformulated to “That it be without physical pain or mainly free from physical pain”, to minimize possible confusion between the concepts of pain and suffering.

## Conclusions

This study contributes to the validation process of the Portuguese version of the Concept of a Good Death scale in the context of GFM. The results demonstrate that the scale has good psychometric properties in this population, revealing a factorial structure consistent with the original model and adequate internal consistency. These findings support the use of the scale to guide clinical practice in primary healthcare, promoting patient- and family-centred care, empathic communication, advance care planning, and coordination with palliative care teams. Additionally, the findings have practical implications, as the scale’s brevity and self-administered format can help raise healthcare professionals’ awareness of individual end-of-life preferences, supporting more person-centred approaches. It may also be incorporated into medical education, particularly in palliative care and communication training, fostering reflection on professionals’ own values and perceptions of death and dying, and contributing to demystifying death within the healthcare community. Overall, the instrument serves as a valuable educational and reflective tool, enhancing communication skills, ethical awareness, and coordination of care, and its use can be further expanded through digital technologies. Further studies are recommended to validate the scale in other professional and cultural contexts and to strengthen its validation through approaches such as CFA.

## Supplementary Information

Supplementary Material 1

Supplementary Material 2

Supplementary Material 3

## Data Availability

The datasets used and/or analysed during the current study are available from the corresponding author upon reasonable request.

## Abbreviations

CFA: Confirmatory Factor Analysis
EFA: Exploratory Factor Analysis
GFM: General and Family Medicine
KMO: Kaiser-Meyer-Olkin
