# Supplementary material for: General and family medicine physicians’ perception of the concept of good death: a contribution to the validation of the scale
Source: BMC Prim Care. 2025 Dec 1;27:5. doi: 10.1186/s12875-025-03113-4 (PMC12777476; doi:10.1186/s12875-025-03113-4)
Supplement: Supplementary file 1 — Supplementary Material 1 [file 12875_2025_3113_MOESM1_ESM.docx]

**Additional file 1.** The Concept of a Good Death measure

| Please indicate how important each of the following is to your conception of a “good” death. | | | | |
| --- | --- | --- | --- | --- |
|  | Not  necessary | Desirable | Important | Essential |
| 1. That it be painless or largely pain-free. | 1 | 2 | 3 | 4 |
| 2. That the dying period be short. | 1 | 2 | 3 | 4 |
| 3. That it be sudden and unexpected. | 1 | 2 | 3 | 4 |
| 4. That family and doctors follow the person’s wishes. | 1 | 2 | 3 | 4 |
| 5. That it occur naturally, without technical equipment. | 1 | 2 | 3 | 4 |
| 6. That it be peaceful. | 1 | 2 | 3 | 4 |
| 7. That loved ones be present. | 1 | 2 | 3 | 4 |
| 8. That the person’s spiritual needs be met. | 1 | 2 | 3 | 4 |
| 9. That the person is able to accept death. | 1 | 2 | 3 | 4 |
| 10. That the person had a chance to complete importante tasks. | 1 | 2 | 3 | 4 |
| 11. That the person had an opportunity to say “good-bye”. | 1 | 2 | 3 | 4 |
| 12. That the person was able to remain at home. | 1 | 2 | 3 | 4 |
| 13. That the person lived until a key event. | 1 | 2 | 3 | 4 |
| 14. That death occurs during sleep. | 1 | 2 | 3 | 4 |
| 15. That there be mental alertness until the end. | 1 | 2 | 3 | 4 |
| 16. That there be control of bodily functions until death. | 1 | 2 | 3 | 4 |
| 17. That the ability to communicate be present until death. | 1 | 2 | 3 | 4 |
