# Supplementary figures and images for: General and family medicine physicians’ perception of the concept of good death: a contribution to the validation of the scale
Source: BMC Prim Care. 2025 Dec 1;27:5. doi: 10.1186/s12875-025-03113-4 (PMC12777476; doi:10.1186/s12875-025-03113-4)

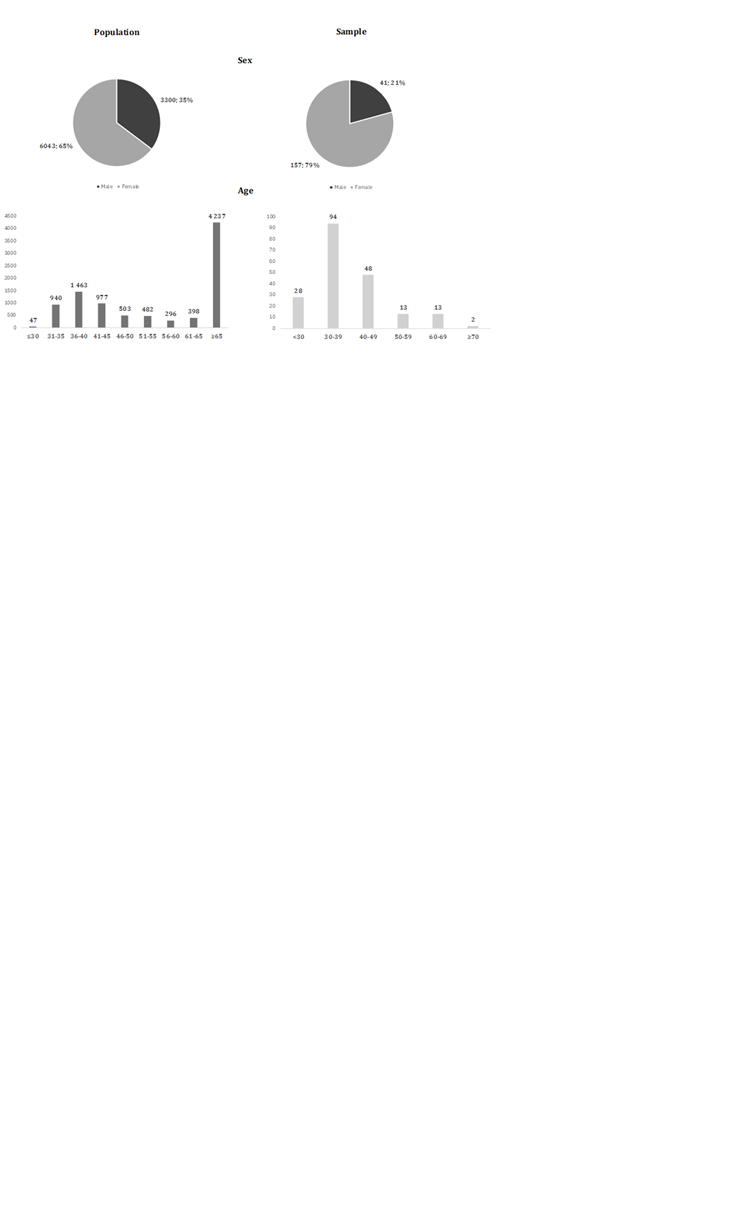

Supplement: Supplementary file 2 — Supplementary Material 2 [file 12875_2025_3113_MOESM2_ESM.png]
