# Supplementary material for: General and family medicine physicians’ perception of the concept of good death: a contribution to the validation of the scale
Source: BMC Prim Care. 2025 Dec 1;27:5. doi: 10.1186/s12875-025-03113-4 (PMC12777476; doi:10.1186/s12875-025-03113-4)
Supplement: Supplementary file 3 — Supplementary Material 3 [file 12875_2025_3113_MOESM3_ESM.docx]

**Additional file 3.** Scale description

|  | **1** | **2** | **3** | **4** | **M (SD))** | **Mdn [Q1; Q3]** |
| --- | --- | --- | --- | --- | --- | --- |
| That it be painless or largely  pain-free. | 2 (1.0) | 9 (4.5) | 29 (14.6) | 158 (79.8) | 3.73 (0.59) | 4 [4; 4] |
| That the dying period be short. | 23 (11.6) | 68 (34.3) | 65 (32.8) | 42 (21.2) | 2.64 (0.95) | 3 [2; 3] |
| That it be sudden and unexpected. | 139 (70.2) | 37 (18.7) | 10 (5.1) | 12 (6.1) | 1.47 (0.85) | 1 [1; 2] |
| That family and doctors follow the person’s wishes. | 1  (0.5) | 19 (9.6) | 42 (21.2) | 136 (68.7) | 3.58 (0.68) | 4 [3; 4] |
| That it occur naturally, without technical equipment. | 70 (35.4) | 83 (41.9) | 31 (15.7) | 14 (7.1) | 1.94 (0.89) | 2 [1; 2] |
| That it be peaceful. | 1 (0.5) | 13 (6.6) | 36 (18.2) | 148 (74.7) | 3.67 (0.62) | 4 [3; 4] |
| That loved ones be present. | 14 (7.1) | 36 (18.2) | 74 (37.4) | 74 (37.4) | 3.05 (0.92) | 3 [2; 4] |
| That the person’s spiritual needs be met. | 2 (1.0) | 16 (8.1) | 51 (25.8) | 129 (65.2) | 3.55 (0.69) | 4 [3; 4] |
| That the person is able to accept death. | 4 (2.0) | 34 (17.2) | 81 (40.9) | 79 (39.9) | 3.19 (0.79) | 3 [3; 4] |
| That the person had a chance to complete important tasks. (n=197) | 0 (0) | 36 (18.3) | 81 (41.1) | 80 (40.6) | 3.22 (0.74) | 3 [3; 4] |
| That the person had an opportunity to say “good-bye”. (n=197) | 1 (0.5) | 22 (11.2) | 67 (34.0) | 107 (54.3) | 3.42 (0.71) | 4 [3; 4] |
| That the person was able to remain at home. | 19 (9.6) | 64 (32.5) | 82 (41.6) | 32 (16.2) | 2.64 (0.87) | 3 [2; 3] |
| That the person lived until a key event. | 13 (6.6) | 68 (34.4) | 75 (37.9) | 42 (21.2) | 2.74 (0.87) | 3 [2; 3] |
| That death occurs during sleep. | 79 (39.9) | 78 (39.4) | 26 (13.1) | 15 (7.6) | 1.88 (0.91) | 2 [1; 2] |
| That there be mental alertness until the end. | 66 (33.3) | 89 (44.9) | 24 (12.1) | 19 (9.6) | 1.98 (0.92) | 2 [1; 2] |
| That there be control of bodily functions until death. | 56 (28.3) | 95 (48.0) | 34 (17.2) | 13 (6.6) | 2.02 (0.85) | 2 [1; 2] |
| That the ability to communicate be present until death. | 32 (16.2) | 91 (46.0) | 49 (24.7) | 26 (13.1) | 2.35 (0.90) | 2 [2; 3] |
